# Concerns and expectations in women with polycystic ovary syndrome vary across age and ethnicity: findings from PCOS Pearls Study

**DOI:** 10.3389/fendo.2023.1175548

**Published:** 2023-08-09

**Authors:** Mirna Elghobashy, Gar Mun Lau, Meri Davitadze, Caroline D. T. Gillett, Michael W. O’Reilly, Wiebke Arlt, PCOS SEva Working Group, Antje Lindenmeyer, Punith Kempegowda

**Affiliations:** ^1^ College of Medical and Dental Sciences, University of Birmingham, Birmingham, United Kingdom; ^2^ Department of Endocrinology and Diabetes, Clinic Neolab, Tbilisi, Georgia; ^3^ Institute of Metabolism and Systems Research, University of Birmingham, Birmingham, United Kingdom; ^4^ Royal College of Surgeons in Ireland (RCSI), University of Medicine and Health Sciences, Dublin, Ireland; ^5^ London Institute of Medical Sciences, Medical Research Council, London, United Kingdom; ^6^ Institute of Clinical Sciences, University of Birmingham, Birmingham, United Kingdom; ^7^ Institute of Applied Health Research, University of Birmingham, Birmingham, United Kingdom; ^8^ Queen Elizabeth Hospital, University Hospitals Birmingham National Health Service (NHS) Foundation Trust, Birmingham, United Kingdom

**Keywords:** polycystic ovary syndrome, qualitative research, education, perceptions, polycystic ovarian syndrome, age, ethnicity

## Abstract

**Objective:**

To understand and explore the perceptions and opinions of women with polycystic ovary syndrome (PCOS) and further delineate the variations across age and ethnicity.

**Design:**

Qualitative survey focussed on lived experiences of people with PCOS. Participants could share their views either as written text or as voice note audio recording(s) on WhatsApp. The data from the audio were transcribed verbatim. Responses were coded by two study members independently, using a thematic inductive method with NVivo 12. Two senior study members then reviewed these codes to identify common themes.

**Subjects:**

Women with PCOS aged 18-60 years.

**Results:**

43 of 45 participants had a formal diagnosis of PCOS, the remaining two had suspected PCOS which was under investigation. Four participants opted to share their views as voice note recordings. Poor mental health was the most reported (83.3% of participants), followed by dermatological (81.0%) and menstrual issues (76.2%). Participants were generally dissatisfied with the care they received (88.1%). A lengthy diagnostic journey was reported in 35.7% of cases. 52.6% felt less feminine, particularly regarding weight gain and infertility. As part of the recommendations by participants, it was emphasised that others with the condition should educate themselves and be proactive in their management. 46.3% reported that being more enlightened regarding their condition improved their health outcomes and enabled them to advocate for their own care. Women in their 20s expressed distress due to poor mental health, needing a longer time to get the diagnosis, and having weight and eating concerns. While women with PCOS in their 30s discussed their menstrual irregularities and fertility issues, those in their 40s expressed their concerns about the societal expectations of women when diagnosed with PCOS. The concerns varied across ethnicities as well.

**Conclusion:**

PCOS has wide-ranging consequences for women living with the condition, with many dissatisfied with the clinical support they currently receive. The concerns and expectations vary across ages and ethnicities. Therefore, we propose involving women with PCOS to co-create clinical and educational resources informed by lived experiences to provide end-user-informed services.

## 1 Introduction

Polycystic ovary syndrome (PCOS) is one of the most common endocrine conditions affecting women of reproductive age with a global prevalence around 10% (1). It classically presents with oligomenorrhoea or amenorrhoea, infertility, hirsutism, acne, and obesity (2). The hallmarks of PCOS include hyperandrogenism, oligo/anovulation and polycystic ovarian morphology. It is also associated with cardio-metabolic diseases such as obesity and diabetes (3), psychological disorders (4) and increased cancer risk (5, 6). The disease is multifactorial, and its complex pathophysiology involves multiple pathways (7). A diagnosis of PCOS is made if other causes of patients’ symptoms are ruled out and patients fulfil two of the three diagnostic criteria (oligomenorrhoea or anovulatory cycles, biochemical or clinical hyperandrogenism, polycystic ovaries seen on ultrasound scan) (8).

PCOS often presents during puberty and the characteristic signs and symptoms coincide with the development of normal physiological findings during this period of life (9, 10), consequently complicating the diagnostic process. Despite its high prevalence, there is still lack of awareness among healthcare professionals regarding its long-term health consequences and its negative impact on mental health (11), consequently leading to delayed diagnosis and treatment. Gibson-Helm et al. reported that a third of women with PCOS reported that it took >2 years and >3 healthcare professionals to establish a diagnosis of PCOS (12). Another multi-methods study has shown that women with PCOS felt they were not taken seriously by their doctors (13). Additionally, women with PCOS often struggle to find reputable sources of information about their condition. Although physicians are integral in the education of patients, the latter often find resources accessed at home much more helpful. It is reported that 98.2% of patients search the internet for PCOS related information (14, 15). A recent systematic review by Lau et al. regarding the experiences of women and individuals with PCOS found that patients struggle to identify relevant, high quality educational resources regarding their condition (16). To date, existing educational and awareness resources for polycystic ovary syndrome (PCOS) have had limited inclusion of patient perspectives. Therefore, we conducted this mixed-methods study to understand the lived experiences, perceptions, and opinions on the current standard of care of women with PCOS which can then help us create a better-represented educational resource for the condition.

## 2 Methods

Women aged 18-60 years were invited to complete an online survey during April and May 2021. This survey was primarily promoted through patient support groups: PCOS Verity, DAISy-PCOS leadership group, and PCOS Vitality. The survey started with information about the study and a privacy statement. Interested participants had to complete an in-built consent form as part of the study before proceeding to answering the questions. Nine closed questions in this survey focused on participant demographics, such as age, gender, ethnicity, country of residence, and history of PCOS diagnosis. Participants with a formal diagnosis of PCOS were asked about the year of PCOS diagnosis, and a further four open-ended questions regarding their lived experiences at the onset of the symptoms of PCOS, their journey during diagnosis, an explanation of PCOS to their younger self, and any advice for their younger self (**Supplementary Table 1**). Participants had the option to share their views for the open-ended questions either as a written text or as a voice note on WhatsApp®. The data from the latter was transcribed verbatim. Phone numbers of participants obtained for collection of voice note data were removed after data collection was complete. All data were anonymised at the point of analysis. Data were then coded using NVivo 12 software and analysed using a combination of content analysis and thematic inductive qualitative methods (17–19). Authors GL and ME read through the data and independently identified and applied codes through open coding. Following this, GL and ME collaboratively organized the codes into themes, revised the codes as appropriate. This process until all data were coded and there was an agreement about the application of codes and themes to the whole data set. Authors PK and AL then reviewed the codes and themes to arrive at an agreement.

After synthesising the themes, we arranged the data into groups based on the participants’ age (20–29, 30–39, 40–49, 50–59) and ethnicity (White and Other). We then studied the frequency of various themes in these groups and highlighted the most commonly occurring themes from them.

## 3 Results

A total of 45 women completed the survey. 43 of 45 participants reported they had a formal diagnosis of PCOS; the remaining two had suspected PCOS which was under active investigation and hence excluded from further analysis. Four participants opted to share their views as voice note recordings; one of them could not be contacted as they had incorrectly indicated their phone number. As a result, data from 42 participants for the open question portion of the survey were analyzed, generating 1326 references towards their experiences and perception about PCOS in total (**Supplementary 2**).

Overall, five common themes emerged: symptoms (504 references by 42 participants), patient journey (421 references by 42 participants), knowledge (197 references by 40 participants), peer-to-peer advice (162 references by 41 participants), and impact of PCOS on social aspects of life (42 references by 19 participants) (**Table 1**). The median and interquartile range of age in the White group was 37 (25.75-41.75) and in the other group was 27 (24–34). The various themes are discussed in greater detail below:

**Table 1:** Thematic data in groups.

		Group					
	Total (n=42)	20-29 yrs (n=13)	30- 39 yrs (n=18)	40-49 yrs (n=6)	50-59 yrs (n=5)	Other races (n=10)	White (n=32)
PCOS symptoms							
	504 references by 42 participants	180 references by 13 participants	215 references by 18 participants	41 references by 6 participants	68 references by 5 participants	108 references by 10 participants	396 references by 32 participants
Mental health	83.3%	92.3%	77.8%	66.7%	100.0%	90.0%	81.3%
Dermatological issues	81.0%	92.3%	72.2%	66.7%	100%	70%	84.4%
Menstrual issues	76.2%	76.9%	83.3%	50%	80%	60%	81.3%
Perception of symptoms	64.4%	76.9%	77.8%	50%	40.0%	70.0%	68.8%
Journey							
	421 references by 42 participants	156 references by 13 participants	175 references by 18 participants	45 references by 6 participants	45 references by 5 participants	93 references by 10 participants	328 references by 32 participants
Attitudes towards healthcare staff	88.1%	84.6%	88.9%	83.3%	100.0%	80.0%	90.6%
Diagnostic experience	71.4%	84.6%	72.2%	50.0%	60.0%	80.0%	68.8%
Management of PCOS	61.9%	53.8%	66.7%	66.7%	60.0%	50.0%	65.6%
Knowledge							
	197 references by 40 participants	88 references by 13 participants	80 references by 17 participants	20 references by 6 participants	9 references by 4 participants	60 references by 10 participants	137 references by 30 participants
Symptoms and signs	50.0%	61.5%	47.1%	50.0%	25.0%	40.0%	53.3%
Hormonal condition	45.0%	69.2%	23.5%	66.7%	25.0%	30.0%	50.0%
Medical terminology	37.5%	46.2%	29.4%	16.7%	75.0%	40.0%	36.7%
Heterogenous presentations	32.5%	46.2%	29.4%	33.3%	0%	50.0%	26.7%
Peer-to-peer advice							
	162 references by 41 participants	47 references by 12 participants	77 references by 18 participants	19 references by 6 participants	19 references by 5 participants	45 references by 10 participants	117 references by 31 participants
Being proactive in own care	48.8%	50.0%	38.9%	50.0%	80.0%	40.0%	51.6%
Becoming educated about PCOS	46.3%	58.3%	50.0%	33.3%	20.0%	70.0%	38.7%
Advice on mentality with a PCOS diagnosis	46.3%	58.3%	44.4%	33.3%	40.0%	70.0%	38.7%
Management of the condition	46.3%	33.3%	55.6%	66.7%	20.0%	70.0%	38.7%
Impact on social life							
	42 references by 19 participants	14 references by 6 participants	15 references by 7 participants	8 references by 3 participants	5 references by 3 participants	11 references by 4 participants	31 references by 15 participants
Societal expectations	52.6%	50.0%	57.1%	66.7%	33.3%	75.0%	46.7%
Everyday life	47.4%	33.3%	42.9%	66.7%	66.7%	50.0%	46.7%
Solidarity with women who have PCOS	42.1%	83.3%	28.6%	0%	33.3%	50.0%	40.0%

### 3.1 PCOS symptoms

The most common symptom participants reported was poor mental health. Within this theme, low mood or depression and a lack of self-worth were most notable. These were frequently discussed in connection with symptoms affecting participants’ outward appearance, particularly weight gain, hair growth, and acne. The second most reported symptom was dermatological issues, particularly hair related problems, namely hirsutism and alopecia, followed by menstrual irregularities. The latter was often the initial symptom prompting to seek professional advice. There were fewer reports of distress associated with this symptom compared to skin and hair concerns, except in the context of fertility. Many participants reported being on a form of contraceptive pill helped alleviate their menstrual irregularity symptoms. Below is an example quotation for PCOS related symptoms. More such examples are included in **Supplementary Table 2**.

Participant 19: “I stress myself out daily just looking at my knickers every time I go to the bathroom just hoping I’ve got a period. Knowing I’m not ovulating and don’t have enough progesterone again makes me feel I’m not a woman and confirms my body is not healthy”.

### 3.2 Patient journey

The most discussed theme within the patient journey domain were attitudes towards healthcare staff. 88.1% felt dissatisfied with PCOS-related healthcare support. Most women felt clinicians were dismissive and lacked empathy. Several participants were confused by their diagnosis and found explanations from clinicians unhelpful.

The second most common theme was diagnostic experience (71.4%). A lengthy diagnostic journey was most frequently reported (35.7%), with many finding it emotionally demanding as diagnosis often took years. Some reported that although clinicians suspected PCOS, their diagnosis was still delayed due to lack of timely investigations, which left women feeling isolated with little professional support.

The third most discussed theme was PCOS management (61.9%). Contraception (40.5%), lifestyle changes (21.4%), and management of dermatological symptoms (21.4%) were most common. Many participants described contraception use in their adolescence for menstrual cycle regulation. The most common contraception used was the oral pill. Lifestyle changes were usually focused on diet rather than exercise, with the aim of weight loss. A number of participants found lifestyle changes stressful. Methods, such as shaving, laser treatment, and using skincare products were reported for management of excess hair growth and acne.

Participant 21: “Terrible. I first went to the doctor about my periods when I was around 19, I had been in bed for a week because of the pain, I was prescribed painkillers, but no further investigation was completed. I went again a couple of years later and was advised that my periods would probably be irregular until I had a baby. I was generally prescribed contraceptive medication without any follow up for the reasons for needing it. In 2018 I went to the doctor to advise that I had come off contraceptive pills and was having issues again with my periods and a GP advised that they suspected PCOS, it then took two years until I had a five-minute appointment with a consultant that confirmed a. I had PCOS and b. There was nothing that I could be given to encourage me to ovulate as I was too fat.”

More examples are included in **Supplementary Table 3**.

### 3.3 Knowledge of PCOS

The most commonly discussed topic in the domain of knowledge of PCOS was signs and symptoms including menstrual symptoms (27.5%), weight and eating (22.5%), dermatological (17.5%), mental health (15.0%) and fertility issues (15.0%).

Some participants used medical terminology when discussing PCOS (37.5%). Notably, the term ‘hirsutism’ was the most frequently used. As expected, participants who employed medical terminologies in their answers, often used it on multiple occasions. Furthermore, 32.5% participants acknowledged the condition’s heterogeneous presentation and clearly understood what a syndrome was and described it accurately.

Participant 7: “… I would say that although the condition involves my ovaries, and that there would be potential implication when trying to get pregnant”.

More examples are included in **Supplementary Table 4**.

### 3.4 Peer-to-peer advice

A concept first established in mental health services, peer-to-peer advice can be defined as a form of social and emotional support that is mutually offered or provided by one person to another sharing a similar health condition to bring about a desired social or personal change (20).

The most common themes in peer-to-peer advice involved being proactive in own care (48.8%), becoming educated about PCOS (46.3%), mentality (46.3%), and management of the condition (46.3%). Many participants recommend being proactive early in one’s PCOS journey. Many considered education important to improve health in the long-term and to advocate for themselves in the future when meeting healthcare professionals. Most advice about mentality centered on remaining positive. Advice on management focused on lifestyle changes, particularly diet and exercise. Many acknowledged that lifestyle changes were not a cure but were associated with better control of the condition and, therefore, improved outcomes.

Participant 31: “It would be to take a driving seat approach, and to put myself at the forefront when it comes to communicating with doctors or telling them what I think is going on with my body”.

More examples are included in **Supplementary Table 5**.

### 3.5 Impact of PCOS on social aspects of life

Within this, the most common theme discussed was the societal expectations of women (52.6%). While all participants identified themselves as women, many believed PCOS rendered them less feminine due to the associated masculine appearance which did not align with current beauty ideals. Some women felt the pressure to bear children.

Another aspect discussed was the condition’s impact on everyday life, including school, career, and interactions with their partners (47.4%). Subfertility issues associated with PCOS were reported to cause strain on romantic relationships, with participants feeling inadequate as a partner.

The third most common theme was solidarity with other women with PCOS (42.1%). Some participants acknowledged the importance of bonding with others with PCOS not only to improve their emotional well-being but also to share experience and gain knowledge.

Participant 7: “I find that the pressures imposed by society on women to be thin and have beautiful hair are in direct opposition with the symptoms I have experienced as a result of PCOS.”

More examples are included in **Supplementary Table 6**.

#### 3.5.1 Concerns and expectations across age groups and ethnicity

More women in their 20s expressed distress due to poor mental health, needing longer time to get diagnosis, and having weight and eating concerns. Interestingly, several women in this age group also acknowledged the condition’s heterogeneous presentation and understood what a syndrome was. While more women in their 30s discussed their menstrual irregularities and fertility issues, those in their 40s expressed their concerns about the societal expectations of women when diagnosed with PCOS.

While women of White ethnicity focused more on the distress due to poor mental health associated with PCOS, women from other ethnicities expressed their concerns with the time it took to get diagnosised and social consequences of the diagnosis.

## 4 Discussion

Seeking patients’ perspectives is vital to improve healthcare experiences and services. While themes regarding symptoms, the patient journey, and knowledge have been reported elsewhere, we report a number of new findings. Our findings emphasise the importance of peer-to-peer advice. Percy et al. found support networks led by specialist nurses to be a significant factor in mitigating the psychological impact of PCOS, especially for newly diagnosed women (21). In the internet era, online support groups have proved to be a versatile and easily accessible source for peer-to-peer support (15). With an ability to remain anonymous and to tap improved to resources from all over the world, online support groups are turning out to be a game-changer in managing chronic conditions like PCOS. Despite this, only 18.8% of patients joined an online PCOS support group or forum (14). It may be beneficial for clinicians to signpost these groups to patients in the future. However, some potential disadvantages should be considered with the use of online support groups, including anxiety because of hearing others’ negative experiences and feelings of being an outsider (22).

Our participants also emphasised the impact of PCOS on their social life. Sharma et al. and Carron et al. highlighted the difficulties women with PCOS encountered in communities where those who struggled to conceive children suffered from devaluation of their social status (23, 24). Some described menstrual symptoms to be debilitating, affecting their school life or career. Similarly, Native American women with PCOS reported being unable to attend tribal ceremonies for fear of unpredictable menses (24).

Women aged 20-29 are more knowledgeable compared to older participants in our study. This may be due to easier access to information on internet and social media. Social media influencers and public events such as PCOS awareness months may also have played a role in engaging the internet-active population (25, 26). Also, taboo may have played a role as topics such as fertility and femininity were not mainstream at the turn of the last century.

Women with PCOS, of non-White ethnicity reported greater delays in diagnosis and discussed mental health and societal pressures more than the rest of the group. To the best of our knowledge, this is the first study of its kind to explore the perception of women with PCOS across age and ethnicity in such detail.

Participants in our study considered some healthcare professionals to be dismissive, lacking clarity in their explanations and empathy. Dissatisfaction with care of women with PCOS may stem from lengthy diagnostic and management processes involved (12, 27) with similar findings reported within other literature (14, 28). PCOS can be difficult to identify due to its heterogenous presentation and presence of several controversies in the pathogenesis, diagnosis, and treatment of PCOS (29). It can also be difficult to manage the symptoms of PCOS once it is diagnosed as only the combined oral contraceptive pill is recommended for the condition. Other medications such as metformin and anti-androgen drugs may also be considered in the management although these are “off-label” uses (1). Longer treatment delay eventually leads to significant periods of time without support or management which can lead to dissatisfaction with care.

Participants in our study reported struggles at the start of their diagnostic journey and following their initial diagnosis of PCOS due to lack of information. Gibson-Helm et al. have reported similar findings, where 60% of women said they were not given information about PCOS when diagnosed, and 20% felt the information given was inadequate (30). Interestingly, the majority of participants in our study demonstrated rather in-depth understanding about PCOS. However, they reported this information was rarely received from healthcare professionals, and instead was a result of their own research.

Mental health was the most experienced symptom in women with PCOS across both non-White (90%) and White ethnicities (81.3%). According to Hillman et al., 74.9% of women with PCOS reported poor mental health due to the condition. Interestingly, they found that women of White ethnic background were more concerned about their mental health and subfertility (43.8% of White participants, compared to 0% non-White participants) than British Asian women (11). This may suggest an area to explore further, to ensure women of all races have adequate healthcare support for mental health and subfertility issues. Additionally, mitigating communication barriers for non-White groups is important to ensure support reaches communities who need it the most.

### 4.1 Strengths and weaknesses

While a sample size of 42 participants may be considered small for quantitative studies, it is considered large for the qualitative methodology we used in the study (31). The rich, detailed data obtained from these 42 participants ensured our ability to perform content analysis and delineate the differences in perception of women with PCOS across age groups and ethnic backgrounds. An average of ~400 words per participant (16,606 words in total) for the four open-ended questions in our survey depicts the long and in-depth answers from participants which adds strength to our findings. Further, all participants in our study came from a verified source, enabling us to confirm the diagnosis of PCOS.

The median time since diagnosis within the participants in this study was 22 years, which is both a strength and weakness; while the lived experience with PCOS over time can help understand the spectrum of PCOS’s impact across the lifespan, our findings may be limited by a recall bias. We also did not have sufficient representation from all the major ethnic groups; hence further studies with large and comprehensive samples are needed to delineate the differences in experience and perception of PCOS across various ethnicities. We acknowledge that the study was conducted during the COVID pandemic and thus opinions and perceptions of PCOS may have been impacted by limited access to clinics and outpatient services.

## 5 Conclusion

Women with PCOS were impacted physically and emotionally by their symptoms and many women felt dissatisfied with the support they received from healthcare professionals. Experiences varied across age and ethnicity further reaffirming the need for individualised approaches when managing PCOS. Therefore, we propose greater involvement of women with PCOS while designing clinical and educational resources to ensure these are acceptable to women living with the condition.

## Data availability statement

The raw data supporting the conclusions of this article will be made available by the authors, without undue reservation.

## Ethics statement

The studies involving human participants were reviewed and approved by Health Research Authority (HRA). The patients/participants provided their written informed consent to participate in this study.

## Author contributions

AL and PK conceptualised the study. ME and GL were involved in all stages of the study since conception, contributing equally to this work and sharing the first authorship. ME, GL, PK and AL conducted the thematic analysis of the data. MD conducted the searches alongside ME and GL and screened the data to ensure appropriateness and authenticity. CDTG provided critical insights during the execution of the study to ensure the voice of public was appropriately captured and recorded in the article. Members of the PCOS SEva team contributed substantially to the conception and design of the study and were involved in discussions at all stages of the study. The PCOS SEva team includes JJC, HKG, MH, SJ, HK, TL, EM, AN, LR, CS, JS, SW, and NZ. AL and PK supervised all stages of the study and share joint senior authorship of this article. ME and GL prepared the original draft of the manuscript. All authors contributed to the article and approved the submitted version.
