# Supplementary material for: Concerns and expectations in women with polycystic ovary syndrome vary across age and ethnicity: findings from PCOS Pearls Study
Source: Front Endocrinol (Lausanne). 2023 Aug 9;14:1175548. doi: 10.3389/fendo.2023.1175548 (PMC10446892; doi:10.3389/fendo.2023.1175548)
Supplement: Supplementary file 1 [file Table_1.docx]

Supplementary Material

Concerns and Expectations in Women with Polycystic Ovary Syndrome vary across age and ethnicity: finding from PCOS Pearls Study

**Mirna Elghobashy^1^, Gar Mun Lau^1^, Meri Davitadze^2^, Caroline D. T. Gillett^3^, Michael W. O’Reilly^4^, Wiebke Arlt^5^, PCOS SEva Working Group^3^, Antje Lindenmeyer^6^, Punith Kempegowda^7, 8^**

1. **College of Medical and Dental Sciences, University of Birmingham, Birmingham, United Kingdom**
2. **Clinic Neolab, Tbilisi, Georgia**
3. **Institute of Metabolism and Systems Research, University of Birmingham, Birmingham, United Kingdom**
4. **Royal College of Surgeons in Ireland (RCSI) University of Medicine and Health Sciences, Dublin, Ireland**
5. **MRC London Institute of Medical Sciences, London, United Kingdom**
6. **Institute of Clinical Sciences, University of Birmingham, Birmingham, United Kingdom**
7. **Institute of Applied Health Research, University of Birmingham, Birmingham, United Kingdom**
8. **Queen Elizabeth Hospital, University Hospitals Birmingham National Health Service (NHS) Foundation Trust, Birmingham, United Kingdom**

*** Correspondence:**Dr Punith Kempegowda

Assistant Professor in Endocrinology, Diabetes and General Medicine

Senior Fellow of the Higher Education Academy

Institute of Applied Health Research, University of Birmingham

Birmingham B15 2TT

Phone: +44 7721 930777

Email: p.kempegowda@bham.ac.uk

**Supplementary table 1: Questions used in the survey**

| **Number** | **Question** |
| --- | --- |
| **1** | I read and understand the survey's information and privacy statement. And I consent to take part in the survey. |
| **2** | What is your year of birth |
| **3** | In order to monitor and improve our diversity, please tell us which gender you identify with? |
| **4** | In order to monitor and improve our diversity, please tell us which ethnicity you identify with? |
| **5** | Are you based in the UK? |
| **6** | Where in the UK are you based? |
| **7** | Which country do you live in? |
| **8** | Have you been formally diagnosed with PCOS? |
| **9** | In which year were you diagnosed with PCOS? (YYYY format) |
| **10** | In which year did you first notice the symptoms of PCOS? (YYYY format) |
| **11** | I read and understand the instructions for the survey |
| **12** | In what format would you like to provide your responses |
| **13** | What is your mobile number including the international dialling code on which we can WhatsApp you? (we will delete the number from our records once we received your response to the survey) |
| **14** | What is your experience till date with the symptoms of PCOS, including their impact on your mood and wellbeing? |
| **15** | What was your experience till date with the process of being diagnosed with PCOS? |
| **16** | Imagine your current self meets your younger self (at the time you first experienced symptoms of PCOS), how would you explain to your younger self what PCOS is? |
| **17** | What advice would you give to this younger self about PCOS, knowing what you know now? |

**Supplementary table 2: Codes and example quotations for ‘PCOS related symptoms’ theme in the survey.**

| **Code** | **Participant** | **Example Quotation** |
| --- | --- | --- |
| **Distressed by PCOS symptoms** | **19** | “I stress myself out daily just looking at my knickers every time I go to the bathroom just hoping I’ve got a period. Knowing I’m not ovulating and don’t have enough progesterone again makes me feel I’m not a woman and confirms my body is not healthy” |
|  | **35** | “PCOS has destroyed my life, my confidence, my hopes and dreams and also has had a major effect on my physical and mental health.” |
| **Low mood/ depression** | **45** | “I was deeply depressed.” |
|  | **14** | “The final symptom that really concerns me is my mental health. I’m a very anxious person and I definitely go through periods of very low mood. I don’t feel that this could be categorised as depression because I’m still able to function at a basic level. But this can last for several days or weeks so it does impact my mood.” |
| **Lack of self-worth** | **15** | “I feel rubbish every time I see myself in the mirror and I worry what other people think of me.” |
|  | **19** | “The lack of confidence and self esteem has never left me no matter how hard I try in altering my appearance, with weight loss.” |
| **Hair related symptoms** | **40** | “I grew a dark moustache at the age of eight.” |
|  | **9** | “I have suffered from hirsutism for many years, it was this that was one of the first symptoms I experienced back in 2011. As time has progressed, the hair has become more of an issue with hair on my chin, chest and lower buttocks/top of thighs”  “creating extra hair which will mean spending more time in the bathroom to get rid of them.” |
| **Irregular menstruation** | **26** | “I have really irregular periods” |
|  | **4** | “periods can be erratic” |

**Supplementary Table 3: Codes and example quotations for ‘patient Journey’ theme in the survey.**

| **Code** | **Participant** | **Quotations** |
| --- | --- | --- |
| **Contraception** | **36** | “put on the pill (dianette).” |
|  | **6** | “before I was on the combined pill I never had a regular cycle, I would go sometimes 8 months without a period and then bleed for a month or two until a doctor would give me medication to stop me bleeding.”  “I then went on Yasmin, which didn't work well for me, then on Gedarel 30/150.” |
| **Lifestyle changes** | **11** | “I. kept on gaining weight again and again until I started to learn more about PCOS and insulin resistance, which made more sense to why I was not losing weight at the beginning. I started changing my methods, gaining gluten/dairy free and trying different alternative. I believe it helped a lot with my symptoms, especially for my gut health as. I used to get bloating very frequently after eating.” |
|  | **45** | I feel like I have gone through some really necessary learning curves e..g diet management after having done all the stupid and dangerous extreme diets under the sun, before finding the safe and sustainable ways to manage things. |
| **Management of dermatological symptoms** | **13** | “I'd had excess hair on my face for years which got me really down. I wasn't given any advice and the only solutions given were some tablets to force an occasional bleed and to go private for any IPL if I wanted to.”  “I always carried tweezers” |
|  | **15** | “Acne was hard for my self esteem and made me feel low. I hated having to use strong creams and take tablets and found the visits to a dermatologist embarrassing.”  “Again my skin is sensitive and attempts to remove the hair can make it look even worse. I can only realise use tweezers and that is time consuming. I end up spending ages in the bathroom and pretend to my family that I am on the toilet. If I have to see someone unexpectedly before I have used tweezers” |
| **Lengthy diagnostic journey** | **21** | “Terrible. I first went to the doctor about my periods when I was around 19, I had been in bed for a week because of the pain, I was prescribed painkillers but no further investigation was completed. I went again a couple of years later and was advised that my periods would probably be irregular until I had a baby. I was generally prescribed contraceptive medication without any follow up for the reasons for needing it. In 2018 I went to the doctor to advise that I had come of contraceptive pills and was having issues again with my periods and a GP advised that they suspected PCOS, it then took two years until I had a five minute appointment with a consultant that confirmed a. I had PCOS and b. There was nothing that I could be given to encourage me to ovulate as I was too fat.” |
|  | **5** | “I first visited the doctors 3 years before getting diagnosed, and I visited several times in between for my irregular periods before they offered me blood tests and an ultrasound scan.” |
| **Dissatisfaction with support from healthcare professionals** | **16** | “Awful. No help, met with lose weight response.”  “Now I’m at a healthy weight I’ve been told there’s nothing to do to help my symptoms. Very frustrated with specialists as you wait months for an appointment for them to not take into considerations my circumstances and only offer advice I could find online.” |
|  | **30** | “I was given a generic leaflet about PCOS and no advice on how to manage my condition and what symptoms to look out for.”  “I was not signposted to any services for support or ways to meet other people with PCOS so at the time I felt very much alone” |

**Supplementary Table 4: Codes and example quotations for ‘knowledge of PCOS’ theme in the survey.**

| **Code** | **Participant** | **Quotations** |
| --- | --- | --- |
| **Description of PCOS: signs/ symptom terms** | **25** | “Weight gain”  “irregular periods”  “your pcos makes it hard for you to lose weight but keep trying you will be ok.”  “Your mood swings and irritability and depression may well be caused by or effected by the syndrome that you have” |
|  | **7** | “hair loss”  “weight gain”  “insulin resistance”  “Finally I would say that although the condition involves my ovaries, and that there would be potential implication when trying to get pregnant” |
| **Description of PCOS: hormonal condition** | **12** | “PCOS is a misnomer - it has less to do with ovaries and more to do with hormones.” |
|  | **31** | “I would explain that PCOS is a complicated condition and that really it’s metabolic and hormonal” |
| **Description of PCOS: constellation of symptoms and presentations** | **41** | a lot of symptoms will come back to pcos . |
|  | **43** | “‘PCOS is an endocrine disorder which is caused by hormonal balance and produces a number of symptoms. The cysts are not the condition, they are a symptom, and you don’t have to have all the symptoms to have PCOS’.” |
| **Use of medical terminology** | **3** | “Hirsutism”  “insulin resistance” |
|  | **23** | “androgenic alopecia” |

**Supplementary Table 5: Codes and example quotations for ‘peer-to-peer advice’ theme in the survey.**

| **Code** | **Participant** | **Quotations** |
| --- | --- | --- |
| **Advice: management of PCOS symptoms** | **18** | “change diet accordingly” |
|  | **22** | “I would say take care of yourself. I would say take care of your health overall, in terms of how you eat and try not to stress about weight, because developing an eating disorder is probably just gonna make it worse.” |
| **Advice: becoming educated about PCOS** | **33** | “The earlier you understand your PCOS, the types and how to manage it better, the better you will be in years to come.” |
|  | **42** | “Try to find out as much as you can about it so that you can look after your health” |
| **Advice: becoming proactive in own care** | **2** | “To seek help early and not to be fobbed off by Doctors who dismiss or try to trivialise your requests for help/treatments.” |
|  | **31** | “It would be to take a driving seat approach, and to put myself at the forefront when it comes to communicating with doctors or telling them what I think is going on with my body” |
| **Advice: Staying positive** | **44** | “Finding an enjoyable lifestyle is so important to help support PCOS symptoms and this is ultimately what will help to find an enjoyable state.”    ‘Don’t be too harsh in yourself and strive for progress not perfection.” |
|  | **30** | “Do not focus on the what ifs. Focus on the now and how you feel currently.” |

**Supplementary Table 6: Codes and example quotations for ‘impact of PCOS on social aspects of life’ theme in the survey.**

| **Code** | **Participant** | **Quotations** |
| --- | --- | --- |
| **Impact on everyday life** | **34** | **“I suffered low moods and also was very isolated when my periods were heavy i tended not to go out much.”** |
|  | **22** | **“the pain can sometimes be really unbearable, and sometimes like you know I’ve been off work because of it. I’ve also been off uni because of it and that’s really really impacted my wellbeing overall”**  **“So I got my periods at around nine, and then I started experiencing like very very heavy periods beyond that, and I think I got to about thirteen or fourteen where it was really unbearable and I couldn’t even you know get through a day of school sometimes because you know I wouldn’t be allowed to go to the bathroom as often, and that, yeah it wasn’t great.”** |
| **Societal expectations of women** | **32** | **“My wellbeing has been impacted: I don't feel as "feminine " as perhaps I might have without the condition.”** |
|  | **7** | **“I find that the pressures imposed by society on womxn to be thin and have beautiful hair are in direct opposition with the symptoms I have experienced as a result of PCOS.”** |
| **Solidarity with other women with PCOS** | **5** | **“And lots of my friends have PCOS, and lots of women worldwide have it too. So there shouldn’t be any shame in speaking up about it, about encouraging research into the area, and if anything I owe it to the women who don’t know or understand they have PCOS to speak up. So the biggest advice to my younger self would be don’t be afraid to talk about it, you’re not alone.”** |
|  | **14** | **“Other women are an incredible help, reach out to people going through what you are. The condition is different for every single person but it’s great to get tips and support from others who understand what you’re going through.”** |
